# Genetic and Sex-Specific Transgenerational Effects of a High Fat Diet in *Drosophila melanogaster*

**DOI:** 10.1371/journal.pone.0160857

**Published:** 2016-08-12

**Authors:** Kelly Dew-Budd, Julie Jarnigan, Laura K. Reed

**Affiliations:** 1 Department of Biological Sciences, University of Alabama; Tuscaloosa, AL, United States of America; 2 School of Plant Sciences, University of Arizona; Tucson, AZ, United States of America; University of Mississippi, UNITED STATES

## Abstract

An organism's phenotype is the product of its environment and genotype, but an ancestor’s environment can also be a contributing factor. The recent increase in caloric intake and decrease in physical activity of developed nations' populations is contributing to deteriorating health and making the study of the longer term impacts of a changing lifestyle a priority. The dietary habits of ancestors have been shown to affect phenotype in several organisms, including humans, mice, and the fruit fly. Whether the ancestral dietary effect is purely environmental or if there is a genetic interaction with the environment passed down for multiple generations, has not been determined previously. Here we used the fruit fly, *Drosophila melanogaster*, to investigate the genetic, sex-specific, and environmental effects of a high fat diet for three generations’ on pupal body weights across ten genotypes. We also tested for genotype-specific transgenerational effects on metabolic pools and egg size across three genotypes. We showed that there were substantial differences in transgenerational responses to ancestral diet between genotypes and sexes through both first and second descendant generations. Additionally, there were differences in phenotypes between maternally and paternally inherited dietary effects. We also found a treated organism’s reaction to a high fat diet was not a consistent predictor of its untreated descendants’ phenotype. The implication of these results is that, given our interest in understanding and preventing metabolic diseases like obesity, we need to consider the contribution of ancestral environmental experiences. However, we need to be cautious when drawing population-level generalization from small studies because transgenerational effects are likely to exhibit substantial sex and genotype specificity.

## Introduction

The effect of environment on an organism’s phenotype has been well documented, however, the effect on descedants has only recently been examined and shows that an ancestor's behavior and environment can affect the health of future generations. Research on human subjects has revealed sex-specific and non-sex specific effects of food resources on multiple generations’ mortality, BMI, and risk of death from diabetes[1,2]. Furthermore, model organisms, such as mice, play a large role in the advancement of this field with studies on the effects on descendant phenotypes of diet[3,4], chemicals [5,6], and trauma [1,2,7,8]. *Drosophila melanogaster* specifically, has been useful in elucidating the transgenerational effects of diet [3,4,9–16], temperature [1,2,5,6,17,18], toxins [3,4,19,20], and immunity [5,6,21,22].

In addition to traditional overall direct transgenerational effects of the environment, we should also consider the role of genotype-by-environment interactions. An extensive genotype-by-diet interaction has been revealed in fruit flies [7,8,23,24] and their offspring [9–13,15,16,25], where some genotypes are highly sensitive to their diets while others are robust to dietary perturbation. The transgenerational aspect of this interaction could indicate either a genotype-specific transmission of dietary information or direct influence of the ancestral phenotypic variation. We would expect to see phenotypic variation among genotypes due to the ancestor’s diet if there is genotype-specific transmission, while a consistent correlation between offspring and ancestral phenotype across genotypes is expected if the effect were strictly due to parental phenotype (*e*.*g*. maternal effect). Maternal effects could be transmitted through the provisioning of the egg by the mother or by maternal loading of transcripts into the egg [26].

The effect of diet on subsequent generations can be quickly and easily modeled in Drosophila, which share basic metabolic functions with mammals, including the insulin/TOR signaling pathway and lipid storage, making them a useful model organism for transgenerational effects of diet [27]. In mammals, studies of epigenetic inheritance must be carried out to the F_3_ generation because the F_1_'s germline is derived *in utero*, thus potentially modifying F_2_ phenotype through grandmaternal (non-epigenetic) effects. However, in Drosophila, studies of genotype-by-diet variation in transgenerational effects need only to be carried out to the F_2_ generation at minimum because Drosophila development occurs outside of the mother, thus maternal effects on an offspring’s germline should be minimal[28,29]. We also have prior evidence that maternal and paternal effects on descendants can vary and should, therefore, be studied separately[29].

Some of the transgenerational dietary studies on Drosophila have revealed the effects of protein deficient [11], protein and sugar deficient[9,10], and sugar-enriched food [13,30] on various metabolic phenotypes, such as offspring size, development time, survival, weight, and metabolic pools. However, these studies have not been able to distinguish between transgenerational effects that are merely a result of a parental phenotype (maternal effect) or are the result of epigenetic inheritance. The distinction between maternal effects or transgenerational epigenetic inheritance is important in determining the mechanism of transmission [31,32]. Maternal effects are a change in offspring phenotype caused by the non-genetic maternal contributions to offspring development, such as egg nutritional content [32]. In contrast, transgenerational epigenetic inheritance indicates effects on offspring phenotype through the non-Mendelian transmission of environmental effects via the gametes leading to alteration of gene expression [31,32].

With these considerations in mind, we sought to determine the genetic variation for sex-specific transgenerational effects of a larval high fat diet. We studied the genotype-by-diet transgenerational effects for male and female pupal weight in ten wild-derived inbred lines [33,34]. We know from previous work that weight exhibits a substantial genotype-by-diet interaction [23,24] and we were interested in whether larger variation in reaction norms can be transmitted across generations, as such effects could influence the long-term health of a population. We then replicated these patterns in a subset of four genetic lines.

We also analyzed three homozygous genetic lines derived from an outcrossed population [35], to determine if patterns we observed in weight also applied to larval triglyceride, trehalose, and protein levels, as well as egg size, of the organisms experiencing either a high fat or normal diet and their descendants. Larval triglyceride (fat storage), trehalose levels (sugar concentration), and protein reflect the metabolic condition of the larva. Assaying these metabolic traits (fly surrogates for metabolic syndrome) can help identify how diet may transmit fitness effects across generations.

In this study, we asked three primary questions. What is the overall effect of genotype and sex of descendants on transgenerational effects (*i*.*e*. do all genotypes react similarly in extent and direction)? Do sex-specific effects occur in the transgenerational impacts on phenotype? And finally, is phenotypic plasticity in the parental generation in response to diet required for future generations to demonstrate transgenerational effects?

## Materials and Methods

### Drosophila stocks

To survey transgenerational weight phenotypes, ten genetic lines, here after referred to as the "***10 line study***" were randomly chosen from the Drosophila Genetic Reference Panel (DGRP); 153, 440, 748, 787, 801, 802, 805, 900, 907, 911, abbreviated as DGRP# [33,34], and from those, a subset of four genetic lines (153, 440, 748, and 911) were selected for further replication, the "***4 line study***", "Fall" referring to the first replicate and "Spring" for the second replicate. For the additional metabolic phenotypes, three stocks were randomly selected (here after referred to as the "***3 line study***") from the Drosophila Synthetic Population Resource (DSPR); 11291, 12062, 22271, abbreviated here as Line A, Line B, and Line C respectively [35,36].

### Experimental design

Flies were maintained in a controlled environment at 25°C on a 12:12 hr light:dark cycle at 50% humidity for the duration of the experiment. Dietary treatments consisted of a standard cornmeal-molasses diet (normal) and an experimental diet (high fat) used in previous dietary studies [23,24,37]. The high fat diet was identical to the normal diet except for the addition of 3% coconut oil by weight before distributing 10 mL of food per vial [23,24]. Prior to experimentation, stocks were maintained on normal food in the controlled environment for more than two generations.

No specific effort was made to control for gut microbiota. Flies acquire their gut microbes from their diet, which is seeded by other flies (*e*.*g*. adults from the previous generation) and environmental sources [38–40]. Lab strains of Drosophila show lower levels of microbial diversity than wild caught flies [41] presumably due, in part, to the combined effects of a sheltered environment, standardized diet, and antimicrobial agents in the food. These genetic lines were maintained in the lab environment and on a diet containing the antimicrobial agents of Tegosept and propionic acid for many generations prior to these experiments. Thus, we expect most of the remaining differences among the genetic lines due to the microbiota are likely to be ultimately under the influence of the genotype of the host and how that genotype filters the small amount of available microbes in lab diet environment.

**Fig 1:**
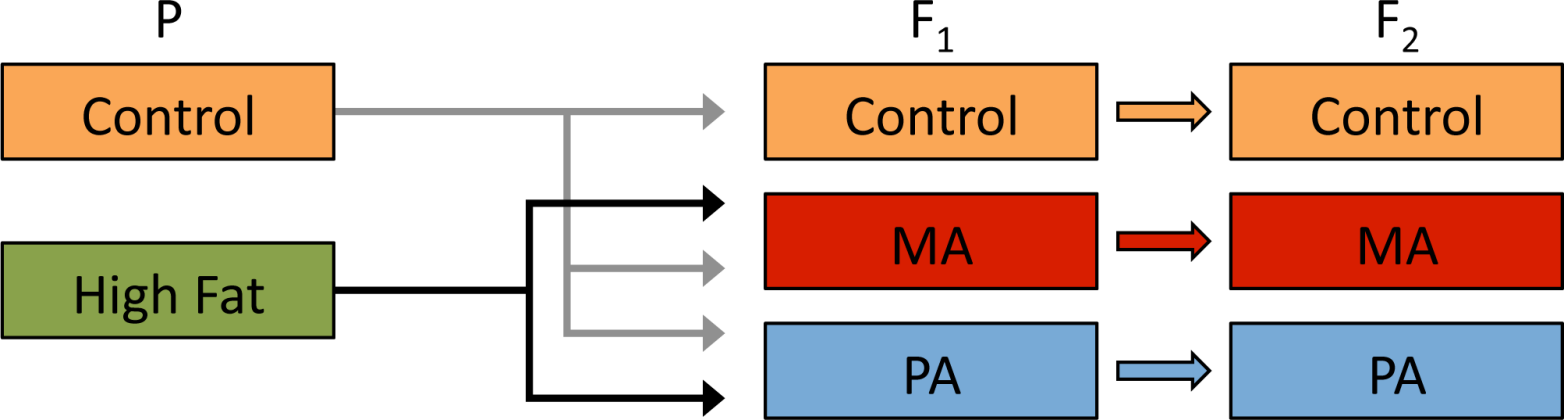
Crossing schematic. In the first generation (P), larvae were fed a normal diet (Control) and a high fat diet (High Fat). P adults within each genetic line were crossed to create three treatment groups for the second generation (F_1_). The Control treatment consisted of both males and females from P:Control. F_1_:MA were offspring from P:Control males and P:High Fat females. F_1_:PA contained offspring from P:Control females and P:High Fat males. F_1_ adults were mated to individuals from the same treatment group to create the third generation (F_2_). All F_1_ and F_2_ larvae were reared on a normal diet.

For each generation, following two days of adult mating, eggs were collected from laying chambers, allowed to hatch, and fifty first-instar larvae were used to seed each food vial. The parental generation (P) was placed on normal food and high fat food. A crossing scheme (Fig 1) was implemented to allow for the distinction of sex-specific transgenerational effects of diet and resulted in three treatment groups, Maternal Ancestor (MA), Paternal Ancestor, (PA) and Control, for two generations (F_1_ and F_2_). The MA treatment group consisted of P females reared on a high fat diet mated to P males reared on a normal diet, while the PA treatment group was the reverse (P males reared on a high fat diet mated to P females reared on a normal diet). Mating between P males and females reared on the normal diet founded the Control lineage.

The F_1_ and F_2_ generations were raised on a normal diet. To prevent the confounding effects of the adult diet in the P generation P pupae were removed from the vials, sexed, and placed in empty bottles until eclosion. Within 12 hours of eclosion, P adults were crossed according to the scheme in Fig 1 for two days on normal food before being placed in laying chambers. The F_1_ generation was allowed to pupate, eclose, and mate for two days within their original food vial, before being placed in laying chambers. The resulting F_2_ larvae were treated identically to the F_1_ larvae. To measure the weight phenotype, at each generation five additional vials were seeded with 50 1st instar larvae and allowed to mature to pupae for collection. For the ***3 line study***, where egg size was measured, several additional vials of F_2_’s were allowed to mature to adults and mate for two days before being moved to laying chambers for egg collection.

Experimental procedures were synchronized within the DSPR and the DGRP genetic lines such that for all genotypes the treated and control groups for a given generation were run simultaneously. This allowed for a robust estimate of genotype-by-diet interaction effect within a given generation, but limited the power of statistical comparisons between generations.

### Phenotypic measurements

All pupae in a vial for the weight phenotypes, known to be quite stable throughout the pupal stage [42], were collected for each generation when they were within 12 hours of eclosion (when they can be sexed), placed in Ringer’s solution, and stored at -20°C. Pupae were cleaned, sexed, and weighed after being patted-dry in groups of three on a high precision balance (Mettler Toledo XS105). In the ***3 line study***, the pupae were weighed individually. We have found that wet weights are much more reproducible than dry weights for the same sample, because for these small masses humidity in the ambient air rapidly rehydrates dried samples skewing measurements.

Fifteen vials from each treatment for each generation were used for quantifying trehalose, triglyceride, and protein levels. Third instar larvae were collected just prior to wandering, pooled across vials, and fasted for 3–4 hours on plain agar plates then flash frozen in liquid nitrogen in groups of ten, with three or more biological replicates per treatment for each phenotype. The larvae were not sexed thus the samples could have exhibited additional variance due to differences in sex ratio. Samples were stored at -20°C until metabolic testing could be completed.

Larval trehalose levels were measured using homogenized larvae and the Sigma Glucose Determination Kit after overnight treatment with trehalase [23,43]. Total larval triglyceride levels were determined in homogenized larvae using the Sigma Triglyceride Determination Kit [23,44,45]. Protein concentrations were determined for the trehalose and triglyceride samples using the AMRESCO Bradford Method Protein Assay Kit [46].

Egg size was measured using 25 randomly selected eggs laid on the first plate retrieved from the laying chambers for a given genetic line and generation. Eggs were measured under a light microscope using a Moticam^®^ 2000. The Motic^®^ Images Plus 2.0 Multilanguage Software package was used to measure the length and width of each egg. Egg volume was then calculated using the formula for an oblate ellipsoid, $\frac{1}{6}\pi a^{2}b$ where *a* is the width and *b* is the length.

### Statistical analysis

Egg size and pupal weight data were normally distributed under a Shapiro-Wilk test. The trehalose and triglyceride data were log transformed, and then normalized for technical effects by calculating residuals from a linear model with a batch effect. For each generation, all normalized phenotypes were analyzed by analysis of variance (ANOVA) according to the model:

$$y=\mu +G_{i}+T_{j}+G_{i}\times T_{j}+E_{ijk}$$

where *G* and *T* are the main effects of genotype and treatment respectively, *G* × *T* is the genotype-by-treatment interaction, and *E* is the error term for the *i*^th^ genetic line, *j*^th^ treatment and *k*^th^ individual. Phenotypes were compared between the two treatment groups (High Fat and Control) for the P generation and the three treatment groups (MA, PA, and Control) for the F_1_ and F_2_ generations. We also conducted additional analyses with more effects such as generation and sex of pupa, and their interactions with genotype and treatment using the same type of ANOVA model. Correlation between the Spring and Fall replicated four genetic lines was calculated on both the average weights within each genotype, treatment, generation, and sex from each replicate, and the differences between treatment (MA or PA) and control averages for each sex, genotype, and generation.

To test the robustness of the p-values determined in the ANOVA, data was permutated 1024 times in Excel. Data was permuted within genotype and sex groups to maintain the genetic and sex based data structure but randomized relative to the F_1_ and F_2_ generations, treatments, and, in the case of the ***4 line study***, replicate, to test for spurious structure in the treatment effects. Each permuted dataset was analyzed by ANOVA and the p-values for all the tested effects were saved. The real p-values were then compared to the distributions of the 1024 permuted p-values for each ANOVA effect.

Post-hoc tests were conducted using Student’s t-test. The correlation between ancestral and descendant pupal weight was determined by calculating the phenotypic difference from Control for the treated ancestor (P or F_1_) of the appropriate sex and the difference between the Control and treated weights in the descendent (F_1_ or F_2_). All statistical analyses were completed using JMP^®^ (version 10.0.0; SAS Institute, Cary, NC). The threshold for statistical significance was p<0.05 unless otherwise noted. Significance values are converted to negative log p-values (NLP) for ease of interpretation in some of the analyses.

## Results

We tested the effect of a high fat diet (3% coconut oil) on the phenotype of future generations by rearing a parental generation (P) on either a normal or high fat diet and then mating them to partners reared on a normal diet (Fig 1). By measuring the following two generations (F_1_ and F_2_), reared on a normal diet, we were able to observe genetic and sex-specific transgenerational effects, as well as, genotype-by-diet interaction effects.

### Weight phenotypes

#### 10 Line Study

**Fig 2:**
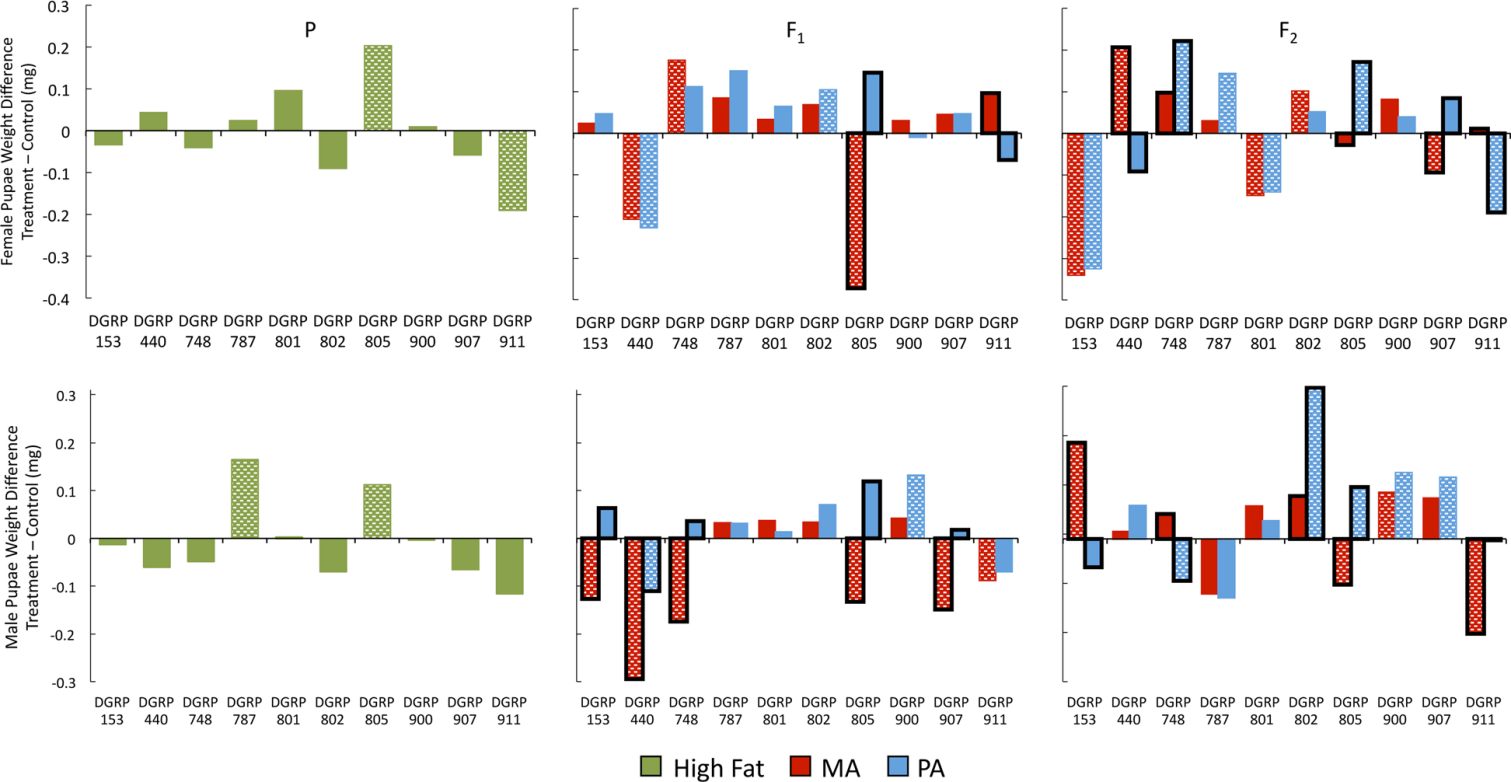
Weight 10 Line Study Data. Polka dots indicate treatment is significantly different from Control. Black border indicates treatments are significantly different from each other. Multiple testing was corrected using false discovery rate of 0.05.

We wanted to explore the genotype-by-ancestral diet interaction effects in a population sample so we measured male and female weights for ten genetic lines from the wild-derived DGRP using the crossing scheme described above (Fig 1). Genotype and genotype-by-treatment effects were highly significant for all generations and sexes tested (Fig 2 and Fig A in S1 Information, Table 1 and Table A in S1 Information). The patterns of variance explained by diet and genotype in the parental generation were very similar to what has been found in previous studies [23,24] where diet alone explained only a very small portion of the total variance but the interaction between diet and genotype explained a substantial portion of the population's phenotypic variance (Table A in S1 Information).

**Table 1:** Descendants (F_1_ and F_2_ generation) variance partition for pupal weight.

Effect^1^	*10 line study* variance explained	NLP^2^	*4 line study* variance explained	NLP
sex	0.3917	312	0.4492	312
genotype	0.1906	312	0.2855	312
treatment	0.0055	13.25	0.0078	18.33
generation	0.0001	*ns*	0.0013	3.91
genotype*sex	0.012	23.02	0.0050	11.02
treatment*sex	0.0014	3.52	0.0010	2.38
treatment*genotype	0.0361	67.03	0.0054	10.03
generation*sex	0.0067	16.96	0.0043	11.18
generation*genotype	0.0145	28.55	0.0051	11.20
generation*treatment	0.0024	5.74	0.0032	7.60
treatment*genotype*sex	0.019	32.83	0.0078	15.39
generation*genotype*sex	0.0077	13.7	0.0018	3.85
generation*treatment*sex	0.0017	4.2	0.0031	7.45
generation*treatment*genotype	0.0157	26.09	0.0069	13.50
generation*treatment*genotype*sex	0.0207	36.27	0.0100	13.40
time replicate	*na*	*na*	0.0119	28.80

^1^ ANOVA model effect

^2^ negative log p-value

*ns*, non-significant

*na*, not-applicable

**Fig 3:**
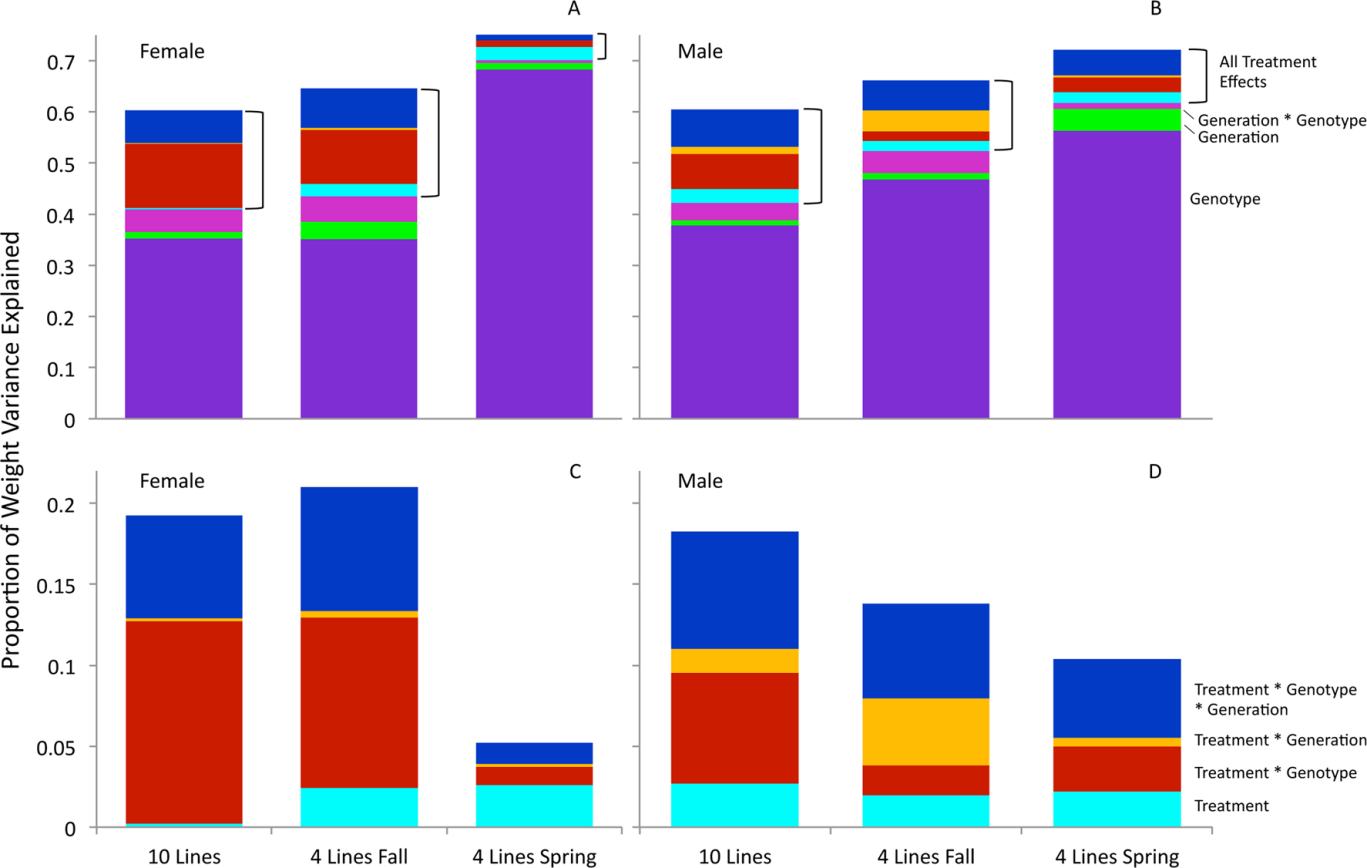
Substantial variance due to treatment effects in F_1_ and F_2_ generations in the partition of variance effects for pupal weight phenotypes. The total explained variance is graphed for females (A) and males (B). The ***10 line study*** is the left most column in each graph while the fall and spring replicates of the ***4 line study*** are the middle and right columns respectively. Variance components are indicated as genotype (periwinkle), generation (green), and generation-by-genotype interaction (fuchsia), while the treatment effects (treatment and all its interactions) are indicated with brackets. The treatment-related effects are enlarged in C (females) and D (males). Variance components of treatment-related effects are indicated as main treatment (aqua), treatment-by-genotype (red), treatment-by-generation (yellow), and treatment-by-genotype-by-generation (blue). The main effect of treatment and its interactions with genotype and generation explain between 5 and 20% of the total variance in the weight phenotype. Relative effect sizes of treatment and treatment-by-genotype-by-generation were consistent across studies in the males but showed more variation in females. The spring replicate of the ***4 line study*** showed reduced total treatment variance effects than observed in the ***10 line study***, and the fall replicate of the ***4 line study***, a subset of the ***10 line study***.

**Fig 4:**
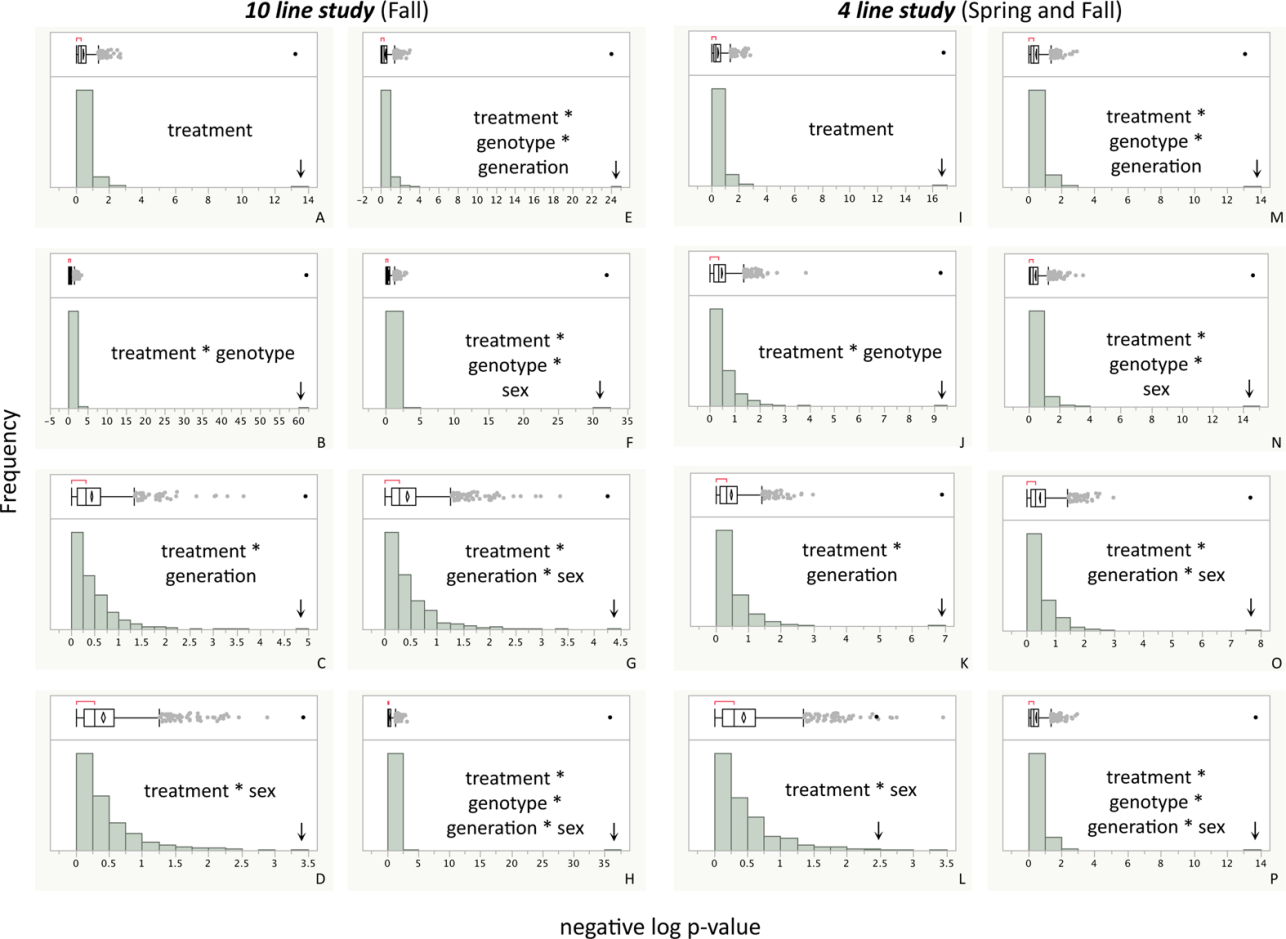
F_1_ and F_2_ generations' distribution of p-values for ANOVA effects with support significance of treatment effects. Arrows indicate actual p-values. Distributions based on 1024 permutations across treatments and generations (and replicate for the ***4 line study***) within genotype and sex to randomize treatment effects. Genotype and sex distributions not shown since they remain highly significant under the permutation model as expected. A-H are derived from the ***10 line study*** while I-P are derived from the Fall and Spring replicates of the ***4 line study***. Note that all real p-values fall in or beyond the extreme tail of the permutation distribution. Additional ANOVA effect permutations can be found in Figs A and B in S1 Information.

When the weight measurements were pooled across the sexes the majority of the variance in weight was explained by sex, genotype, and genotype-by-treatment, with slight contributions from genotype-by-sex and genotype-by-treatment-by-sex (Table 1 and Table A in S1 Information). The total variance contributed by the sex-by-treatment interaction and those interactions with genotype and generation in the F_1_ and F_2_ generations was 4% (Table 1). There was no significant contribution of generation alone to weight, but there were highly significant interactions between generation and treatment, genotype, and sex in the F_1_ and F_2_ generations (Table 1). When considering the main treatment effect and all of its interactions with other factors in the F_1_ and F_2_ generations, those effects were responsible for 18%-19% of the variance in phenotype within each sex (Fig 3, S1 File). We tested for the robustness of the strong interaction effects found in the ANOVA by performing permutation tests that randomized across treatments and generations within sex and genotype for the F_1_ and F_2_ generations. All p-values for the interaction effects fell well beyond the distribution of randomly generated p-values showing the analysis to be robust to random factors (Fig 4A–4H and Fig B in S1 Information).

#### 4 Line Study

We replicated the effects of ancestral diet on pupal weight in four genetic lines selected from the original ***10 line study***. We found the overall patterns to be very similar with larger contributions of genotype and sex to the total variation in pupal weight, and small contributions of treatment interaction effects (Fig C in S1 Information, Table 1 and Table A in S1 Information, S1 File). The total contribution of treatment and its interaction effects to variation in weight within the sexes accounted for more than 20% of the total variance for females and 14% for males in the Fall replicate but it was smaller in the spring replicate at about 5% and 10%of the total variance for females and males respectively (Table 1 and Table A in S1 Information, Fig 3, and S1 File).

The main effect of treatment remained consistent across all studies in the males, as did the relative contribution of treatment-by-genotype-by-generation (Fig 3D). For females, there were more substantial differences in the relative effect sizes of treatment and treatment's interaction effects across the three studies (Fig 3D). While the relative contribution of treatment-by-genotype and treatment-by-genotype-by-generation were consistent in the ***10 line study*** and the subset of the genetic lines in ***4 line study*** fall replicate, those factors were much less substantial in the spring replicate of the ***4 line study*** for females (Fig 3C). The main effect of treatment however, did remain consistent across the Fall and Spring replicates of the ***4 line study***.

**Fig 5:**
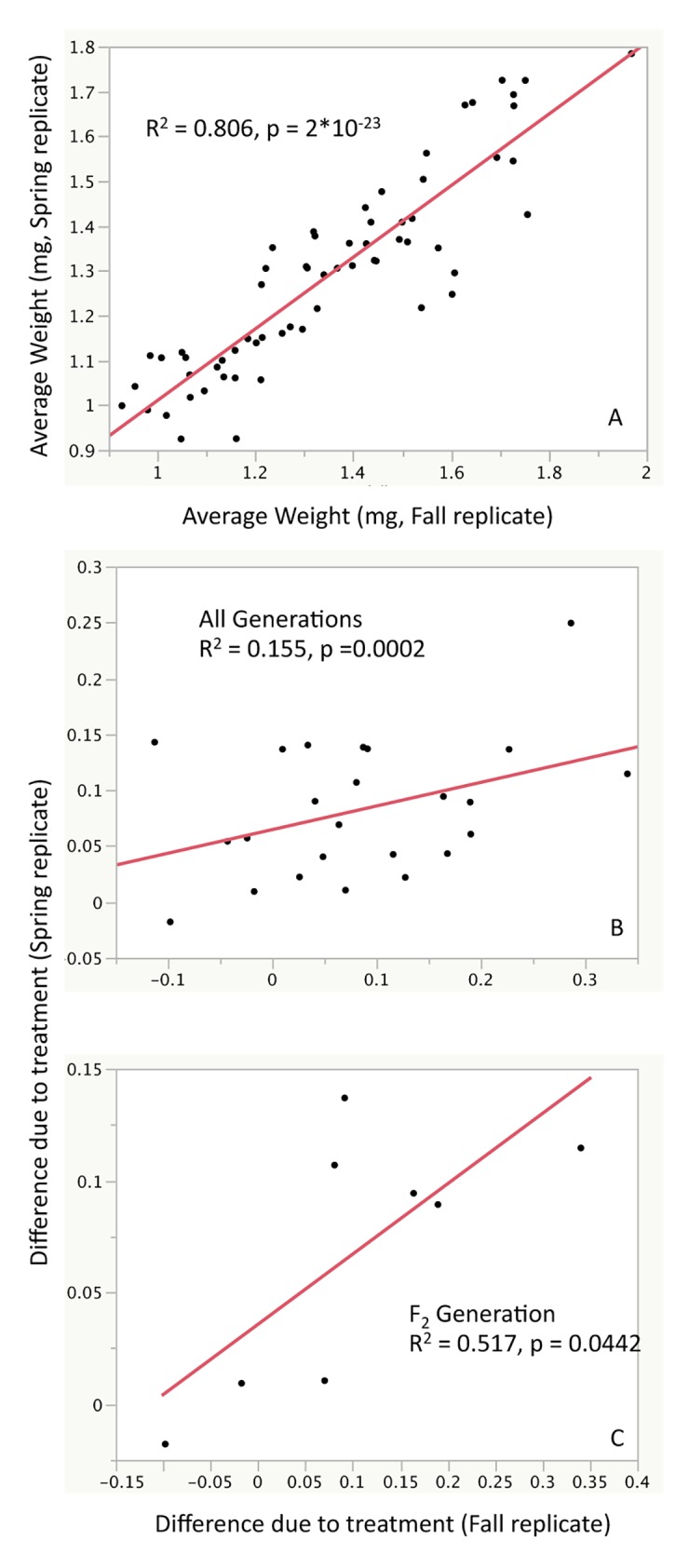
Significant correlation between the Fall and Spring replicates of the *4 line study*. The overall correlation of the actual measurements for all three generations across four genetic lines and three treatments and two sexes showed robust correlation (A). The difference between the average control and treated measurements, across replicates within genotype/sex/generation combinations, also shows a significant positive correlation. (B). When the F_2_ data points are isolated from the total set in (B), the correlation is stronger and significant (C).

The overall correlation between the Fall and Spring replicates was greater than 80% (Fig 5A). When the major effects of genetic line and sex were controlled for (by taking the difference between the control and treated sample averages for each sex/genotype/generation) the positive correlation between the two replicates remained significant (p = 0.002) with an R^2^ value of 0.155 (Fig 5B). In addition, when permutation tests were performed randomizing across the spring and fall replicates, treatment, and generation within genotype and sex, the actual p-values fell to the extreme right tail of the null p-value distribution indicating that the sex and genotype specific treatment effects are robust across study replicates.

#### F_2_ generation reaction norms

**Fig 6:**
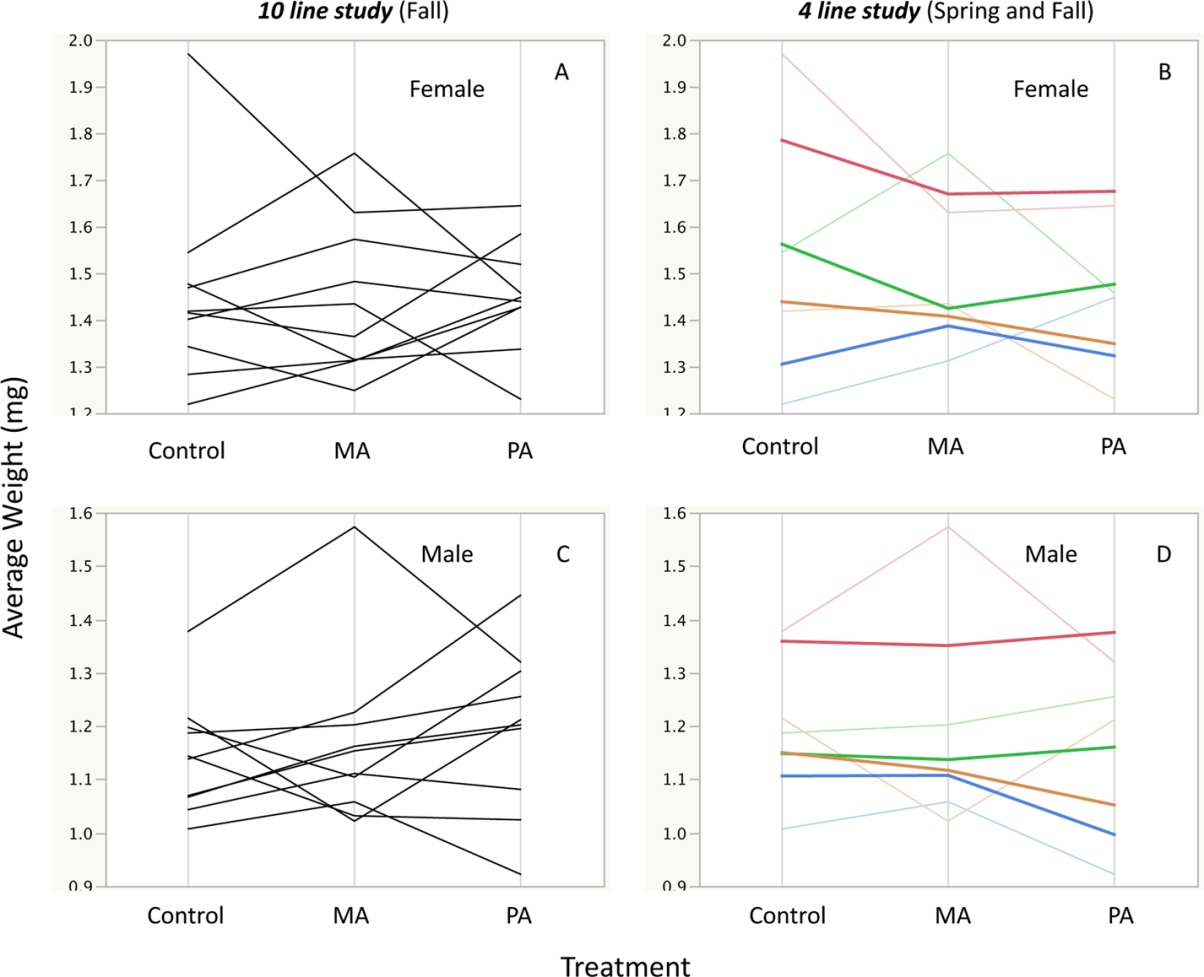
Reaction norms for pupal weight in the F_2_ generation. The left panels indicate the 10 reaction norms across the three treatments for female pupal weight (A) and male pupal weight (C). The right panels indicates the subset of four genetic lines from the original 10 in the Fall and their Spring replicates for female (B) and male (D) pupal weight, bold indicates the reaction norm from the Spring replicate. For most genetic lines, the reaction norms across the two time replicates in panels B and D maintained a similar pattern.

We are especially interested in how the diet of the grandparents (P generation) affects the phenotypes of the grandchildren (F_2_ generation). The reaction norms across the control, MA, and PA treatments varied dramatically across the ten genetic lines first tested (Figs 2 and 6), and the total variation due to genotype-by-treatment interactions in the F_2_ generation explained 22% of the variance in female weight (NLP = 46.6) and 17% of the variance in male weight (NLP = 46.7) (Fig 3). The large effect of the P generation treatment was specific to the sex of the P ancestor experiencing the treatment for some genetic lines (*e*.*g*. 911 females, Figs 2 and 6, Table 2) and for other genotypes the treatment effect was independent of the sex of the ancestor (*e*.*g*. 153 females, Figs 2 and 6, Table 2). In addition, the magnitude and direction of the effect of the ancestral diet varied by genotype and by sex of the weighed pupae (e.g. 802 males and females, Fig 2). The sex-by-treatment-by-genotype interaction effect was highly significant and explained over 6% of the total variance in the F_2_ generation (NLP = 52.4, S1 File).

**Table 2:** Significant independent contrasts in F_2_ generation.

		*10 line study*										*4 line study*^a^			
		153	440	748	787	801	802	805	900	907	911	153	440	748	911
female	Control vs PA	*	*	*	*	*		*		*	*	*	*		*
female	Control vs MA	*	*	*		*	*		*	*		*	*	*	
female	MA vs PA		*	*				*		*	*		*	*	*
male	Control vs PA	*	*	*	*		*	*	*	*				*	*
male	Control vs MA	*		*	*	*	*	*	*	*	*				
male	MA vs PA	*		*			*	*			*			*	*

^a^ ***4 Line Study*** from Spring Replicates

* indicates significance at p< 0.05

In the four genetic lines that were retested, their F_2_ reaction norms followed a fairly similar pattern (Fig 6B and 6D). The correlation between the differences of the control and treated samples in the F_2_ generation was positive and substantial with an R^2^ of 0.517 (p = 0.0442, Fig 5C). Permutation tests were performed on the ***4 line study*** weight data where sample measurements were randomized across replicates, generations, and treatments within each genetic line and sex for the F_1_ and F_2_ generations. All p-values for the ANOVA effects for the real data fell in or beyond the extreme tail of the p-values calculated from permuted data indicating that the highly significant effects of treatment interacting with genotype, sex, and generation are not due to random chance (Fig 4I–4P and Fig B in S1 Information).

#### Predictive power of ancestral treatment phenotype

We also tested the predictive power of the ancestral phenotypic difference to the MA or PA treatment on future generations (Table 3). Correlations were calculated between reactions to dietary treatment rather than raw weight to control for experimental variation between generations for the ***10 line study***, the Fall replicate of the ***4 line study*** (subset of the ***10 line study*** data), and the Spring replicate of the ***4 line study*** (Table 3). Any differences in correlation between the ***10 line study*** and the Fall replicate of the ***4 line study*** demonstrate how a subset of a population may differ from the whole, while differences between the Fall and Spring replicates of the ***4 line study*** demonstrate how correlations can breakdown across time in the same genetic sample.

**Table 3:** Predictive power of ancestral phenotype on descendant pupal weight.

Generation^1^	Sex^2^	Treatment^3^	Ancestor Effect^4^	*10 Line* Correlation^5^	*4 Line* Correlation Fall^6^	*4 Line* Correlation Spring^7^	*10 Line* P-value^8^	*4 Line* P-value Fall^9^	*4 Line* P-value Spring^10^	Repeatability^11^
F1	F	MA	P_HF_diff F	**-0.59**	**-0.48**	**-0.20**	**1.28E-19**	**2.31E-06**	**3.38E-02**	*
F1	F	PA	P_HF_diff M	**0.19**	**0.27**	-0.51	**1.07E-02**	**1.44E-02**	1.18E-08	*x*
F1	M	MA	P_HF_diff F	-0.02	**-0.49**	**-0.28**	*ns*	**2.21E-06**	**3.28E-03**	
F1	M	PA	P_HF_diff M	**0.28**	**0.54**	-0.49	**3.14E-04**	**1.90E-07**	3.08E-07	*x*
F2	F	MA	P_HF_diff F	-0.05	0.06	0.04	*ns*	*ns*	*ns*	
F2	F	PA	P_HF_diff M	**0.25**	0.16	**0.22**	**1.36E-04**	*ns*	**2.31E-02**	
F2	M	MA	P_HF_diff F	-0.27	0.58	-0.06	5.90E-04	4.21E-06	*ns*	
F2	M	PA	P_HF_diff M	**-0.23**	**-0.22**	**-0.26**	**7.12E-04**	**2.68E-02**	**8.70E-03**	*
F2	F	MA	F1_MA_diff F	**0.02**	**-0.05**	-0.36	***ns***	***ns***	1.24E-04	
F2	F	MA	F1_MA_diff M	0.01	-0.60	-0.11	*ns*	6.78E-07	*ns*	
F2	F	PA	F1_PA_diff F	**0.46**	**0.41**	-0.42	**9.17E-13**	**1.18E-05**	6.46E-06	*x*
F2	F	PA	F1_PA_diff M	**0.40**	**0.30**	0.03	**1.05E-09**	**1.50E-03**	*ns*	
F2	M	MA	F1_MA_diff F	0.24	-0.05	-0.10	1.68E-03	*ns*	*ns*	
F2	M	MA	F1_MA_diff M	-0.12	-0.01	0.16	*ns*	*ns*	*ns*	
F2	M	PA	F1_PA_diff F	0.02	-0.48	0.24	*ns*	5.41E-07	1.71E-02	
F2	M	PA	F1_PA_diff M	0.21	**-0.43**	**-0.25**	2.23E-03	**8.33E-06**	**1.39E-02**	*x*

^1^ generation of weighed pupae

^2^ sex of weighed pupae

^3^ Treatment—MA (maternal ancestor on high fat), PA (paternal ancestor on high fat)

^4^ generation (P, F_1_), treatment (HF, MA, PA), and sex of the difference between control and treated ancestor

^5^ correlation between ancestor and progeny phenotype in the ***10 line study***

^6^ correlation between ancestor and progeny phenotype **in 4 line study** subset of ten genetic lines

^7^correlation between ancestor and progeny phenotype in ***4 line study*** replicated

^8^ P-value of ***10 line study*** correlation

^9^ P-value of **4 line study** subset of ten genetic lines

^10^ P-value of ***4 line study*** replicate.

^11^ repeatability across populations and time points (* correlation repeats in all three studies, *x* one of the studies contradicts the pattern repeated in the other two studies), *ns—*non-significant, bold indicates correlation repeated in at least two studies

In general, pupae weight differences in the F_1_ generation tended to be negatively correlated with the MA treatment effect, but positively correlated in the PA treatment (Table 3). In the F_2_ generation, there was no consistent pattern of the magnitude of the parental treatment effect, but females showed more positive correlations with the F_1_ treatment effects and males showed more negative correlations. However, the patterns of correlation across generations was highly variable across studies, while ten of the 16 tested correlations replicated in at least two of the studies, only two were consistently negatively correlated across all three studies, one each in the F_1_ and F_2_, while four of the correlations that replicated in two studies were substantially contradicted in the third (Table 3).

### 3 Line Study metabolic phenotypes

We were interested in whether the patterns observed in weight were also reflected in other phenotypes including trehalose, triglyceride, and protein levels, male and female pupae weight, and egg size. These phenotypes were measured in the P, F_1_ and F_2_ generations for three randomly selected genetic lines from the DSPR.

#### Metabolic pools

Genetic diversity contributed significantly to variation in protein concentration for all three generations, to trehalose in the F_1_ and F_2_ generations, and to triglycerides in the P and F_1_ generations (Table 4). Treatment group contributed to protein levels for the P and F_2_ generation, and triglycerides in the P generation (Table 4). Protein levels also showed a significant genotype-by-treatment interaction in the F_1_ generation (Table 4). The genotype-by-treatment effect in the F_2_ generation explained 2.8% of the variation in triglycerides, 6.0% of the variation in trehalose, and 6.2% of the variance in protein (S1 File). For Line A, larvae exposed to a high fat diet had higher trehalose levels (*p* = 0.0284), higher protein levels (p<0.0001), and lower triglyceride levels (*p* = 0.0003) than those on the control diet (Fig D in S1 Information, Table 5). Additionally, Line A F_1_:MA had higher protein levels than F_1_:PA (Fig D in S1 Information, p = 0.0329) and F_2_:MA had lower trehalose levels than F_2_:Control (Fig D in S1 Information, Table 5, *p* = 0.0497). Line C showed an increase in protein in the MA and PA treatments in the F_1_ generation (p = 0.0193 and p = 0.0051 respectively) and a decrease in protein in the MA treatment relative to both the PA (p = 0.0037) and control (p = 0.0347) treatments in the F_2_ generation (Fig E in S1 Information). Line B did not show significant differences between treatments for triglyceride, trehalose, or protein levels. Our study pooled male and female larvae for the metabolite measurements, but future studies of transgenerational effects on larval metabolite pools may want to consider whether the sex of the larvae plays a role.

**Table 4:** P-values of ANOVA effects for each phenotype separated by generation. Significant *p*-values are bolded.

	Trehalose Levels			Triglyceride Levels			Female Pupal Weight		
Comparison	P	F_1_	F_2_	P	F_1_	F_2_	P	F_1_	F_2_
Genotype	*ns*	**3.71**	**3.27**	**3.33**	**3.17**	*ns*	**2.47**	*ns*	**7.89**
Treatment	*ns*	*ns*	*ns*	**2.37**	*ns*	*ns*	*ns*	**1.48**	**1.41**
Genotype * Treatment	*ns*	*ns*	*ns*	*ns*	*ns*	*ns*	*ns*	*ns*	*ns*
	Protein			Egg Size			Male Pupal Weight		
	P	F_1_	F_2_	P	F_1_	F_2_	P	F_1_	F_2_
Genotype	**5.97**	**2.47**	**1.42**	**3.22**	*ns*	**11.21**	**4.22**	*ns*	**2.29**
Treatment	**3.17**	*ns*	**1.39**	**1.40**	*ns*	*ns*	*ns*	**3.45**	*ns*
Genotype * Treatment	*ns*	**2.21**	*ns*	*ns*	*ns*	**1.76**	*ns*	**3.22**	*ns*

**Table 5:** Significant differences between treatment groups for each phenotype/generation/genotype.

	Trehalose Levels ^b^			Triglyceride Levels ^b^			Female Pupal Weight ^b^		
Comparison ^a^	P	F_1_	F_2_	P	F_1_	F_2_	P	F_1_	F_2_
MA vs. Control	A*	-	A*	A***	-	-	A*	-	A* C *
PA vs. Control		-	-		-	-		A**	C**
MA vs. PA		-	-		-	-		A**	-
	Protein ^b^			Egg Size			Male Pupal Weight ^b^		
	P	F_1_	F_2_	P	F_1_	F_2_	P	F_1_	F_2_
MA vs. Control	A***	C*	C*	B*	-	-	-	-	-
PA vs. Control		C**	-	-	-	C**		-	-
MA vs. PA		A*	C**	-	B**	A* C**		-	-

^a^ Only significant comparisons are listed. A significant difference is represented by the genotype’s letter designation, the level of significance, and the directionality of the difference. A bold entry indicates ‘x’ was a larger value in the comparison ‘x vs. y’. (t-test; *p* < 0.05 *, *p* < 0.01 **, *p* < 0.001 ***)

^b^ The only comparison made in the P generation was High Fat vs. Control.

#### Male and female pupal weight

Male and female pupal weight both had genotype effects for the P and F_2_ generations (Table 4). Ancestral diet (treatment) caused significant effects in the F_1_ and F_2_ generations for female weight (Table 4), and these effects were driven by Line A and Line C (Fig F in S1 Information, Table 5).

#### Egg size

**Fig 7:**
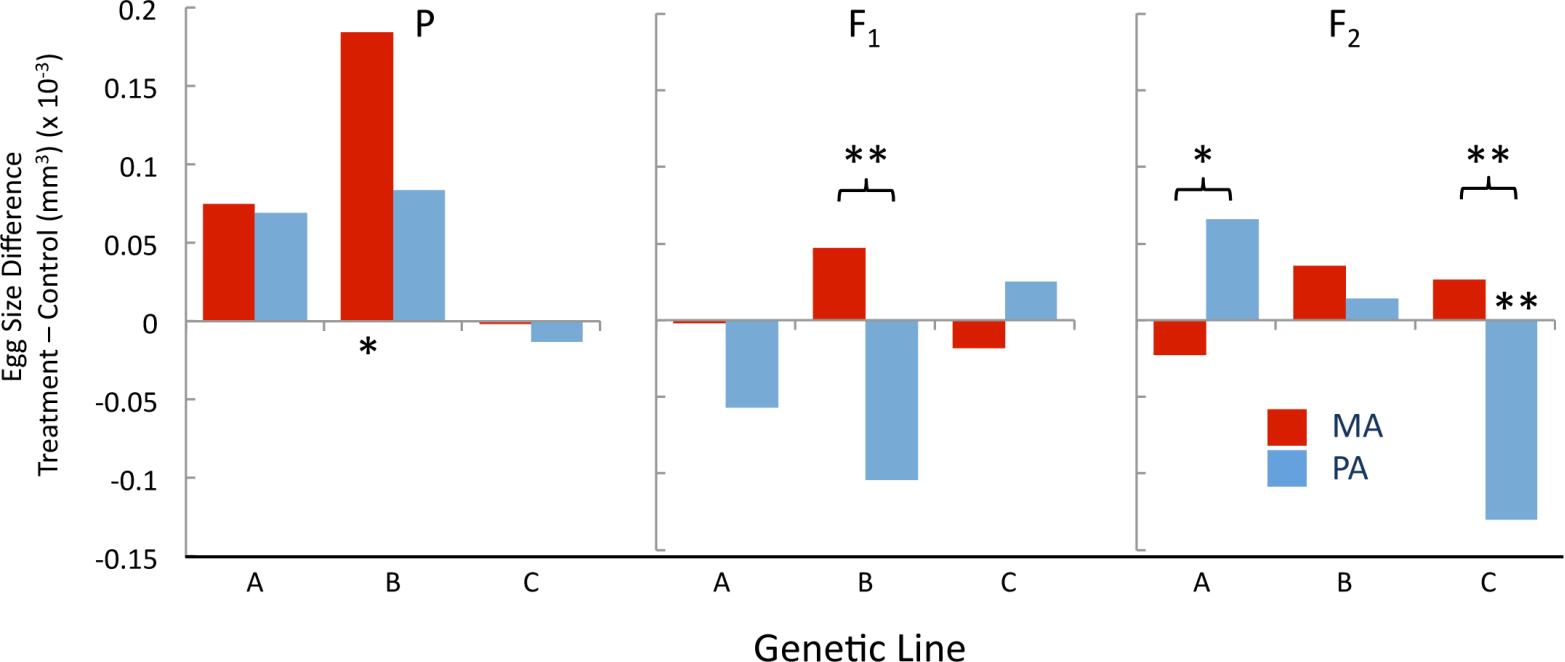
Difference in egg size of Maternal Ancestor (MA) and Paternal Ancestor (PA) compared to the Control in the *3 line study*. Substantial differences by sex, genotype, and generation were observed for the influence of the high fat treatment. Significance values indicated as *p* < 0.05 *, *p* < 0.01 **.

The P and the F_2_ generations had a large genotypic effect on egg size (Table 4). As well, egg size was affected by treatment in the P generation and genotype-by-treatment interaction explained 4.2% of the variation in the F_2_ generation (Table 4). Interestingly, the genotype-by-treatment effect is caused by Line A and Line C’s opposing reactions to the treatments (Fig 7 and Fig G in S1 Information). Line B eggs laid by a female who was reared on a high fat diet (P:MA) were 8.3% larger than the Control (Fig 7 and Fig G in S1 Information, Table 5, *p* = 0.0206). Line B eggs laid by a female with a mother reared on a high fat diet (F_1_:MA) were 7.2% larger than the F_1_:PA eggs (Fig 7 and Fig G in S1 Information, Table 5, *p* = 0.0054). Line A F_2_:PA had 4.1% larger eggs than the F_2_:MA (Fig 7 and Fig G in S1 Information, Table 5, *p* = 0.0423). Opposingly, Line C’s F_2_:PA had smaller eggs than F_2_:MA (7.1%, *p* = 0.0013) and the F_2_:Control (Fig 7 and Fig G in S1 Information, Table 5, 5.9%, *p* = 0.0067).

#### Egg-body size correlation

We also looked at the correlation between male and female pupal weight and egg size (Table B in S1 Information). Overall, treatment groups with large male pupae laid larger eggs (*p* = 0.0004). This relationship was driven by genotype-specific relationships and Line B’s positive correlation between pupal size and offspring’s egg size (*p* = 0.0194). Line B’s female pupal weight was positively correlated with the size of the eggs they laid (*p* = 0.0113). In contrast, Line C’s female pupae had a negative correlation with laid egg size (*p* = 0.0311). Interestingly, there was a negative correlation between Line B’s egg size and the future male and female pupal weights (*p* = 0.0262 and 0.0469, respectively).

## Discussion

In this study we examined the sex-specific effects of a high fat diet on two subsequent generations in the model organism, *D*. *melanogaster*. We have demonstrated three important patterns about transgenerational epigenetic effects of a diet: 1) differences in ancestral diet exhibit a significant interaction with genotype and sex in descendants' phenotype, 2) epigenetic impacts on phenotype vary according to the sex of the affected ancestor, 3) ancestral reaction to a difference in diet is not a consistent predictor of a descendants' weight.

### Substantial genotype and sex-dependent transgenerational effects

Our findings were consistent with previous studies that investigated only one generation past treatment (F_1_) compared to two in our study, in that each genotype showed a unique array of transgenerational effects [13]. We found that the genotype-by-treatment interaction effect in both the F_1_ and F_2_ generations for weight was highly significant and explained 1–22% of the total variation in phenotype within a given generation and sex. To put this into perspective, effect sizes for quantitative trait loci deemed important for influencing the genetic component of human phenotypes and disease through GWAS are routinely on the order of 1% [47]. Thus, the influence of ancestral diet interacting with genotype appears to be as important, and most cases much more important, than the effect of individual genetic loci. Further study with many more replicates will be needed to make a robust estimate of the absolute effect of the interaction between ancestral diet and genotype, but clearly is a non-negligible effect.

Interactions involving the sex of the descendant and the diet of the ancestor are also highly significant in the F_1_ and F_2_ generations, explaining 2.5 and 6.7% of the total variation in weight in each generation in the ***10 line study***, and 1.0 and 3.1% of the variation respectively in the ***4 line study***. Overall, when considering F_1_ and F_2_ in combination, 5–10% of the variance in weight was due to the parent generation's diet (treatment) and its interactions with the descendants' genotype, sex, and generation (Table 1). The importance of treatment interactions with genotype and generation are even more apparent when the sexes are analyzed separately, with the main effect of treatment and all of its interactions explaining between 5 and 21% of the phenotypic variance (Fig 3).

We observed similar magnitudes of treatment effects in the phenotypes measured in the ***3 line study***, suggesting that the transgenerational impact of ancestral diet can influence multiple traits, including ones closely tied to fitness such as egg size. Xia and Belle [25] also recently found that adult diet can effect longevity and reproduction out as far as the F_3_ generation in a single genotype of Drosophila. Our findings of treatment effects across generations is consistent with previous research that has found that the dry weight of F_1_ adults whose parents were fed a high sugar [13] or low protein diet [11] were heavier than the controls. It should be noted that the variance explained by the main effect of ancestral diet is not directly additive to the interaction of the treatment with other factors such as sex or genotype, thus it is possible that the treatment can have an average directional impact across all samples, but also have a distinct effect on one sex or genotype.

### Sex-specific transmission

Considering all measured phenotypes, maternal and paternal ancestors did not always affect descendants to the same magnitude. In many of the instances where an ancestral treatment of one sex (MA or PA) varied from Control, the other ancestral sex high fat treatment does not differ from Control (Table 2). For example, in the ***10 line study***, the MA treatment in the F_2_ generation had no significant impact of female pupal weight but was associated with a substantial decrease in weight in the male pupae. Conversely, the PA treatment was associated with a decrease in the weight of female F_2_ pupae but not in male F_2_ pupae. Overall, the MA and PA treatments differed from each other in F_2_ pupal weights within sex for five of the ten genetic lines each for females and males (Table 2). For seven out of the eight contrasts between MA and PA in the F_2_ generation in the ***4 line study***, the pattern replicated (Table 2). Similar patterns were observed for the other phenotypes in the ***3 line study***. Thus, the sex of the grandparent experiencing a distinct dietary treatment influences the nature of the transgenerational impact on the grand-offspring.

In humans, Kaati *et al*. [1] found that transgenerational dietary influences occurred only through the paternal line in their human cohort, where the father and paternal grandmother’s exposure to famine during their slow growth period was associated with lower cardiovascular causes of death in the present generation. The paternal influence may be transmitted via DNA methylation and small non-coding RNA expression in the spermatozoa of obese males [48]. Our data does not show clear indications of strictly paternal (PA) inheritance, and instead there are effects through both the paternal and maternal lineage.

In addition, a previous study in Drosophila found that a maternal (MA) high sugar diet affected the metabolic pools of both male and females in a specific genetic line for two generations [30]. In our experiment, one of the three genetic lines tested for changes in metabolic pools showed MA high fat treatment effects on trehalose levels in the F_2_ generation (Table 5). Interestingly, paternal low protein diet did not affect female body size in a previous study of Drosophila [11], but paternal high fat diet did affect female size in a mouse model [49]. Temperature effects on body size in Drosophila have also been observed to transmit in a sex-specific manner, where mothers who developed at higher temperatures had offspring with a decreased body mass, while the fathers that develop at higher temperatures produced offspring with a longer wingspan [17]. Taken as a whole, these results show that sex-specific multi-generation effects can occur, but will vary by genotype.

### Parental reaction an inconsistent predictor of descendants’

The impact of the diet on the parental phenotype may be due to differences in feeding rate between the two diets and/or due to inherent nutritional differences of the diets. For the purpose of the questions posed in this study, which focus primarily on whether the environmental experience of the parent generation impacts the F_1_ and F_2_ generations, we do not need to know the underlying mechanism of the diet effect in the parental generation to be able assess how difference in the parental environment impact subsequent generations. However, future studies on how the added fat effects palatability would be interesting since it would allow us to differentiate between the impact of diet composition and total calorie consumption on subsequent generation's phenotypes.

The population showed a high degree of variation to an ancestral high fat diet and this may indicate that the transmission of transgenerational effects is rooted in genotype-specific mechanisms and less in broadly applied maternal effects, thus genetic mapping may be a useful way to identify how genetic variants affect transgenerational inheritance, and thus identify the underlying genetic mechanisms that contribute to these patterns. However, the moderate correlations observed between generations for pupal weight across ten genetic lines implies that there may be some general predictions about transgenerational effects at the population level. Correlations in phenotype across generations in a genetically variable population implies that there is an aspect to the inheritance mechanism(s) that can function independently of the genotypes of the individuals, such as through environmentally determined epigenetic modification.

Similarly, in a previous study, glucose levels in females fed a high sugar diet were not changed but the offspring in the F_1_ and F_2_ generations had higher glucose levels than the control for the one tested genotype [30]. This indicates a parental reaction to diet is not required for descendant phenotypic effects, which may be an example of direct induction in Drosophila [50]. We saw a number of genetic lines where the treatment in the parental generation had little or no effect on the phenotypes of the parents, but the F_1_ and F_2_ descendants showed a substantial impact from the parental diet (Fig 2).

Certainly, explorations of potential mechanisms for transgenerational inheritance within genetically variable populations must be sensitive both to genotype-dependent and genotype-independent factors (such as sex and other environmental factors). For example, for female weight we found that the main effect of the parental treatment was negligible overall in the ***10 line study*,** but the subset of four genetic lines assayed in the Fall showed a more substantial main effect of treatment that replicated in the spring as well (Fig 3C). Thus, the shift in the genetic population analyzed changed the overall variance due directly to the ancestral generation's diet. Overall, the predictability of ancestral diet is largely genotype-specific and future studies should utilize large genotypic sample sizes to accurately characterize a population's general transgenerational phenotypic effects and avoid attributing a genotypic idiosyncrasy to population-level patterns.

In a genotype-specific context, another explanation for the "skipped" generational dietary effects is Mendelian inheritance of high fat exposed alleles. Due to independent assortment of high fat diet exposed alleles in the F_2_ generation into four possible epigenetic "genotypes," we might have expected to see an increase in variance in phenotypes in F_2_ generation for the treated relative to control if there was Mendelian inheritance of additive transgenerational epigenetic effects. However, genotypes with phenotypic responses only in later generations did not show a consistent increase in variance for the F_2_:MA and F_2_:PA compared to F_2_:Control, and there was no effect of generation or treatment on the within-genotype variation. Thus, two identical-by-decent alleles from the high fat exposed grandparent did not apparently amplify the phenotypic response in the F_2_ generation, implying that epigenetic effects are not, categorically, additive.

Finally, we found that correlations between egg size and pupal size were genotype-specific, confirming previous findings of no consistent effects of egg size on adult weight or offspring egg size across a population, which may be due to accumulation of environmental effects interacting with genetic variation [51].

## Conclusions

In this study, we found that there was only a moderate link between parental reaction to a high fat diet and their untreated descendants' phenotype, and the associations exhibited extensive genotype-specific reactions. This indicates that there is a potential for deleterious effects to occur in descendants even when the ancestor did not demonstrate a negative reaction to the environmental stress. Additionally, a transgenerational effect in one sex was not a reliable indicator of effects in other related traits. For example, female pupae were affected in some genetic lines while males were not affected (Fig 2).

Sex-specific treatment and phenotyping across generations has shown its usefulness in Drosophila, humans, and mice, where the two sexes can perform in dramatically different ways. Sex-specific effects may offset each other and prevent researchers from detecting transgenerational environmental impacts if the sexes are not analyzed separately. Future studies should focus on the elucidation of the sex-specific effects by parsing treatment groups further (*e*.*g*. high fat treated maternal grandfather) and phenotyping males and females separately. Additionally, the additive effect of sex should be considered in organisms whose ancestors of both sexes were raised in a high fat background, as in Valtonen *et al*. [11]. These additional tests will help determine the origin, and thus mechanism, for the transgenerational transmission of dietary information.

Finally, the dramatic differences between all genetic lines show the importance of testing multiple genotypes to assess the genetic diversity of a transgenerational trait, since not all genotypes react to the same extent or direction. Epigenetic effects only evaluated in a single genetic background may lead to inaccurate conclusions because of genetic variation in a real population. Awareness of genetic variation for the impact of ancestral environment could provide important insights into the varied causes of the current obesity epidemic.

## Supporting Information

S1 File Supplemental Data Tables. Tables of data means and standard errors, raw data, and variance partitions under different models are given.
(XLSX)

S1 Information Supplemental Figs and Tables. Figs A-G and Tables A and B can be found in the S1 Information file. Figs A and B give distribution of P-value permutations, Fig C graphs the differences between control and treated flies for the ***4 line study***, while D- G depict genotype-, generation-, and treatment-specific means and standard errors for measured phenotypes. Table A gives ANOVA variance partition for the parental generation. Table B gives the correlation between body weight and egg size.
(PDF)
