# Supplementary material for: Genetic and Sex-Specific Transgenerational Effects of a High Fat Diet in Drosophila melanogaster
Source: PLoS One. 2016 Aug 12;11(8):e0160857. doi: 10.1371/journal.pone.0160857 (PMC4982694; doi:10.1371/journal.pone.0160857)
Supplement: S1 Information — Figs A-G and Tables A and B can be found in the S1 Information file. Figs A and B give distribution of P-value permutations, Fig C graphs the differences between control and treated flies for the 4 line study, while D- G depict genotype-, generation-, and treatment-specific means and standard errors for measured phenotypes. Table A gives ANOVA variance partition for the parental generation. Table B gives the correlation between body weight and egg size. (PDF) [file pone.0160857.s002.pdf]

Supplemental Information: Genetic and Sex-specific Transgenerational Effects of a High Fat Diet in *Drosophila melanogaster*

Kelly Dew-Budd[1,2], Julie Jarnigan[1], and Laura K. Reed[1*]

[1] Department of Biological Sciences, University of Alabama; Tuscaloosa, AL, USA [2] School of Plant Sciences, University of Arizona; Tucson, AZ, USA

*Corresponding author

E-mail:lreed1@ua.edu (LKR)

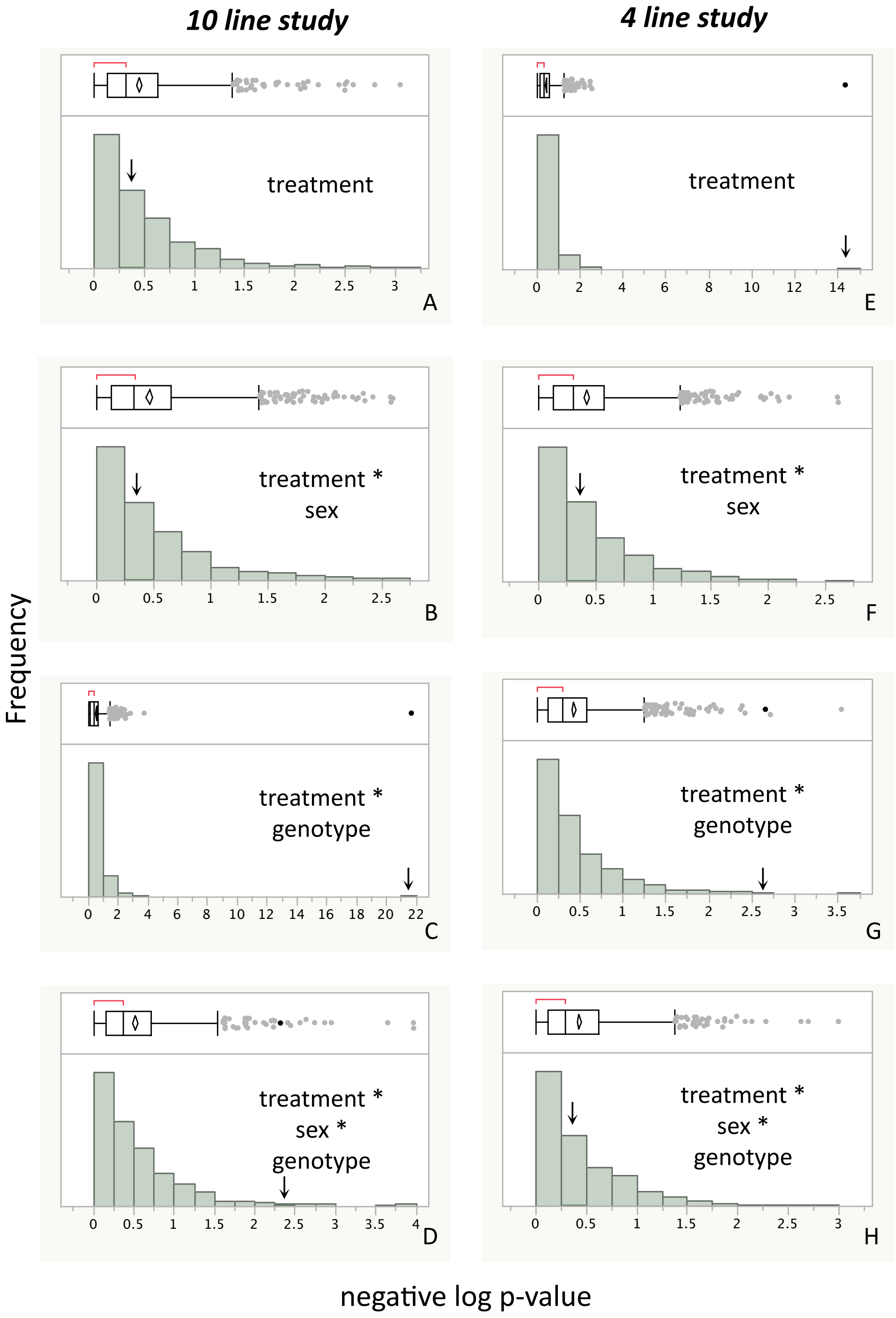

Figure A. Parental generation distribution of p-values for ANOVA effects with permutation support significance of treatment effects. Arrows indicate actual p-values. Distributions based on 1024 permutations within genetic line and sex to randomize treatment effects. Genetic line and sex distributions not shown since they remain highly significant under the permutation model. A-D are derived from the ***10 line study*** while E-H are derived from the fall and spring replicates of the ***4 line study***. A and E are treatment main effects, B and F are the treatment interaction with sex, C and G are the treatment interaction with genetic line, and D and H are the three way interaction between treatment, genetic line, and sex.

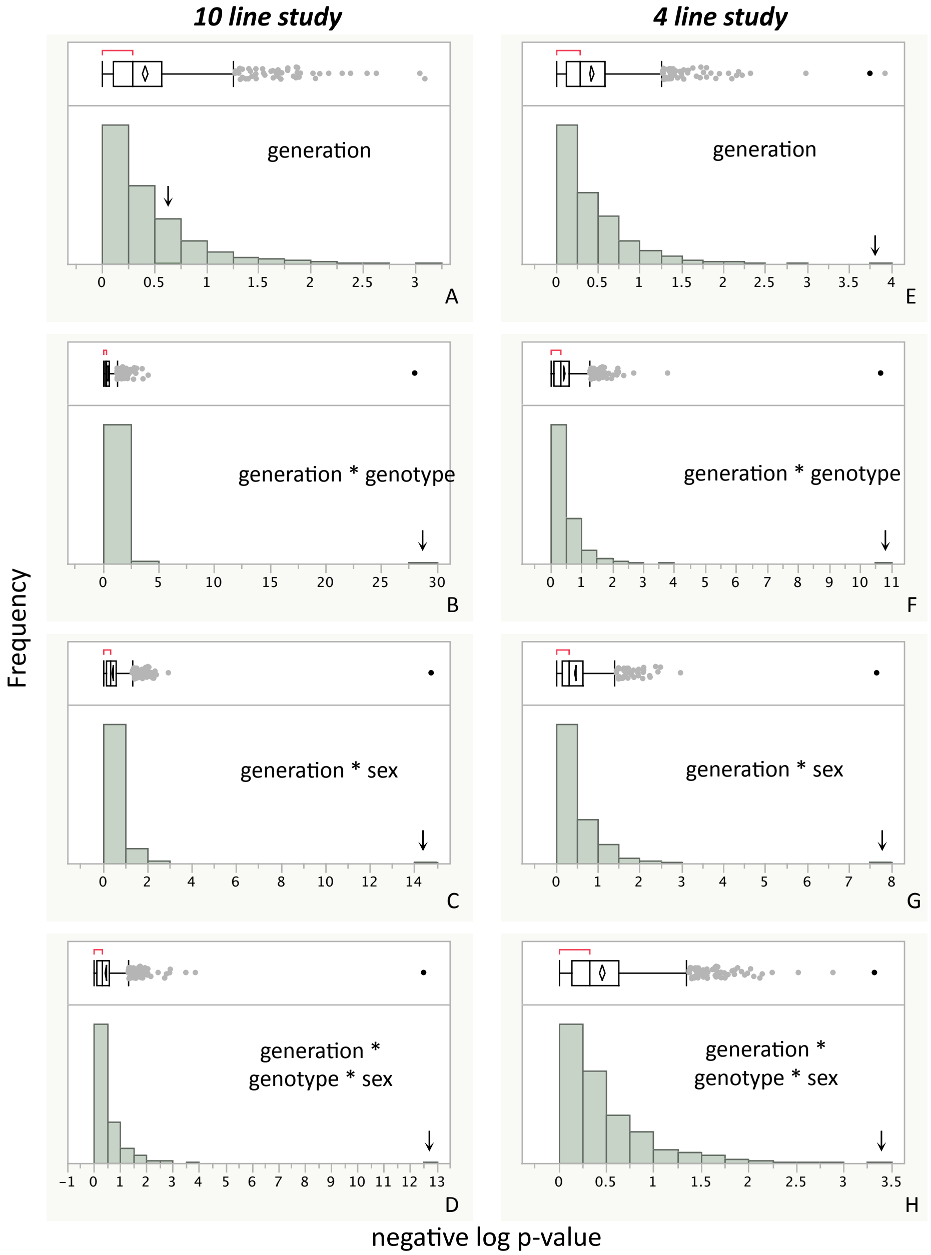

Figure B. F1 and F2 generation distribution of p-values for ANOVA effects with permutation support significance of effects other than treatment. Arrows indicate actual p-values. Distributions based on 1024 permutations within genetic line and sex to randomize treatment effects. Genetic line and sex distributions not shown since they remain highly significant under the permutation model. A-D are derived from the ***10 line study*** while E-H are derived from the fall and spring replicates of the ***4 line study***. A and E are generation main effects, B and F are the generation interaction with genetic line, C and G are the generation interaction with sex, and D and H are the three way interaction between generation, genetic line, and sex.

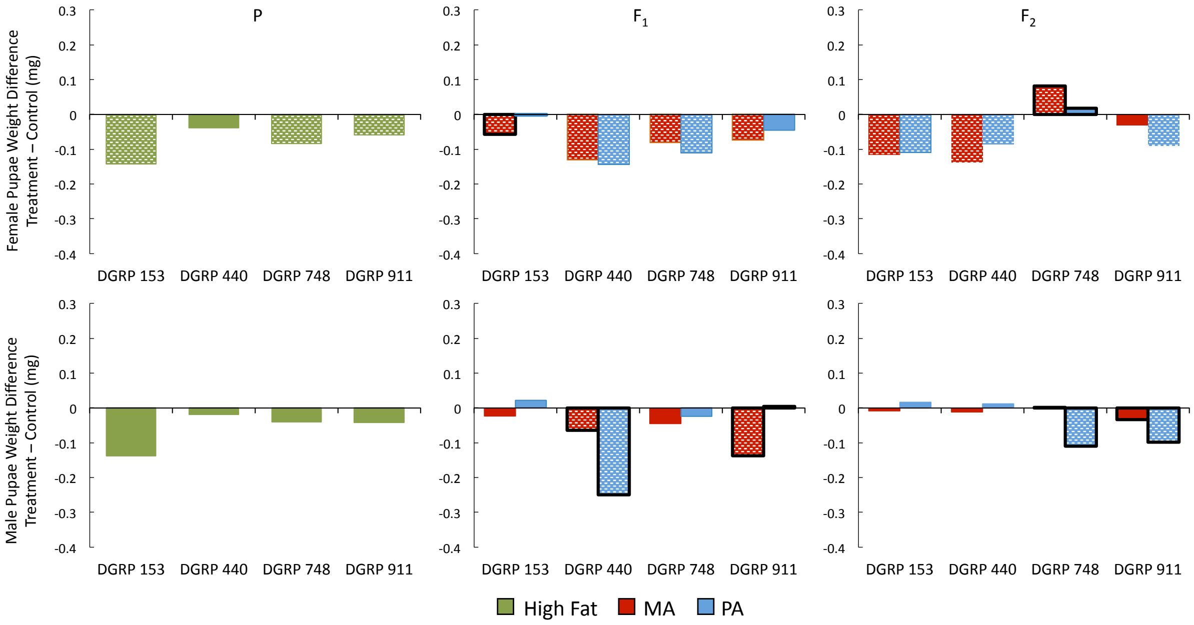

Figure C. Weight *4 Line Study* Data. Polka dots indicate treatment is significantly different from Control. Black border indicates treatments are significantly different from each other. Multiple testing was corrected using false discovery rate of 0.05.

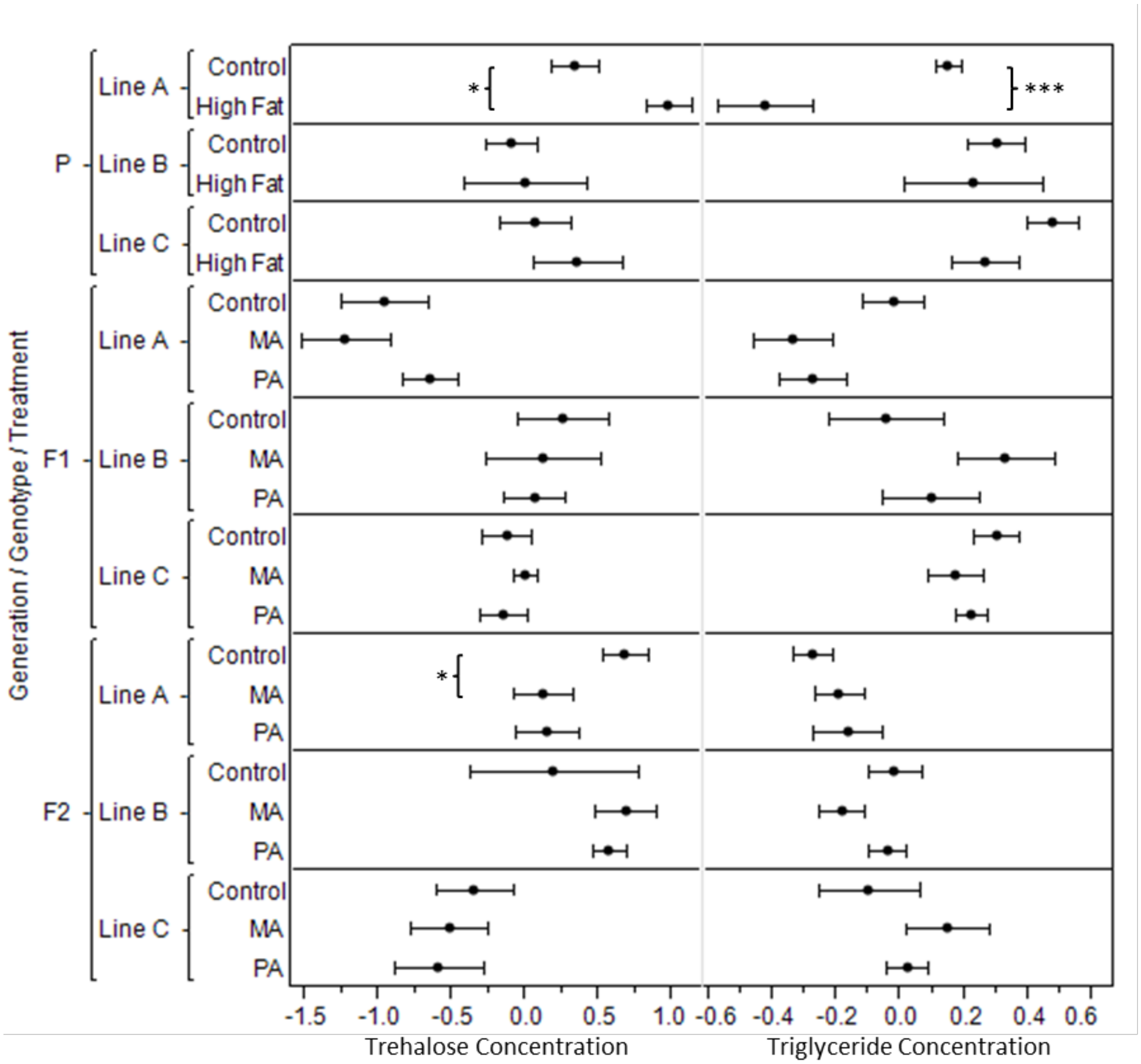

Figure D. Trehalose and triglyceride concentrations (mean ± SE) of Control, High Fat, Maternal Ancestor (MA), and Paternal Ancestor (PA) for three generations. Raw data was log transformed and the residuals for batch effects are graphed. Triglycerides were corrected for protein concentration of the sample. Significance values indicated ($p < 0.05$ *, $p < 0.01$ **, $p < 0.001$ ***).

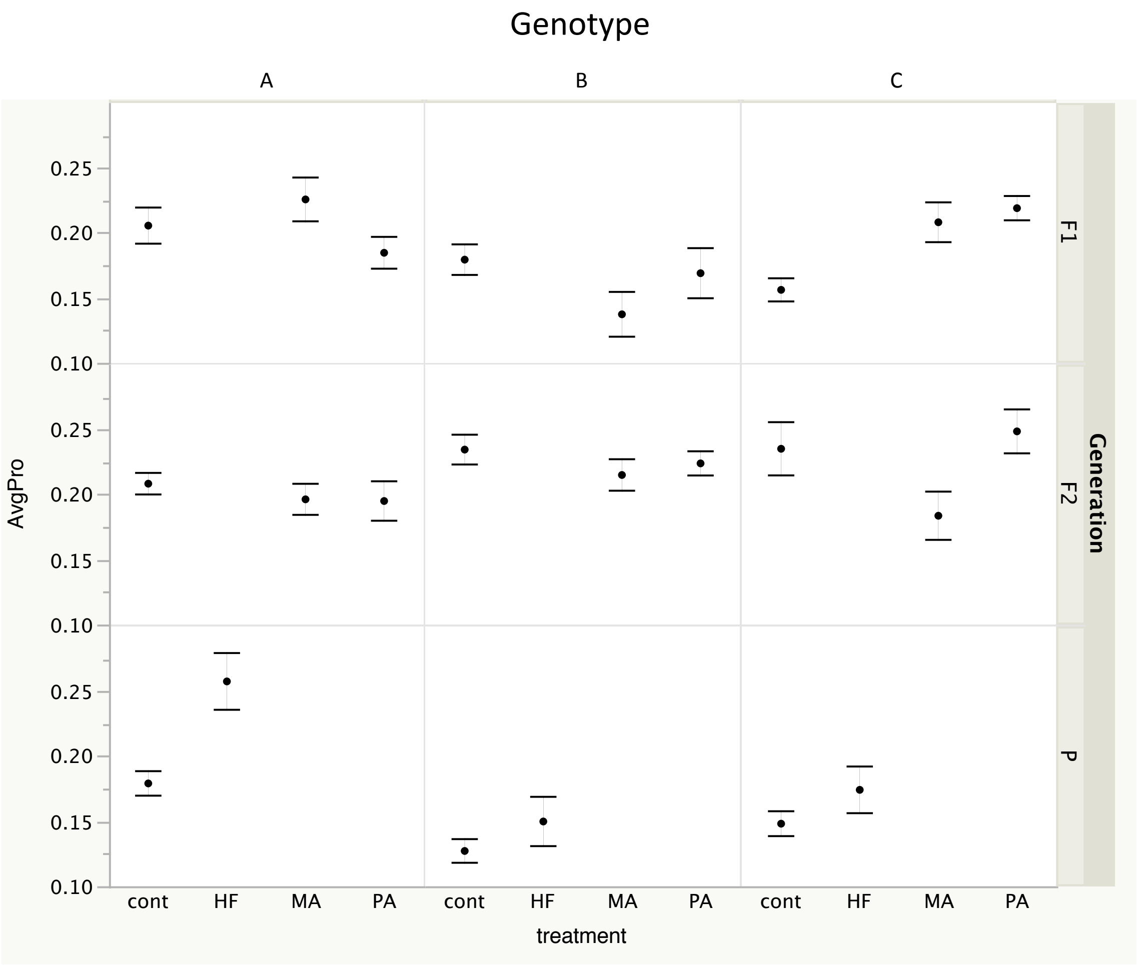

Figure E. Protein concentrations (mean ± SE) of Control, High Fat, Maternal Ancestor (MA), and Paternal Ancestor (PA) for three generations.

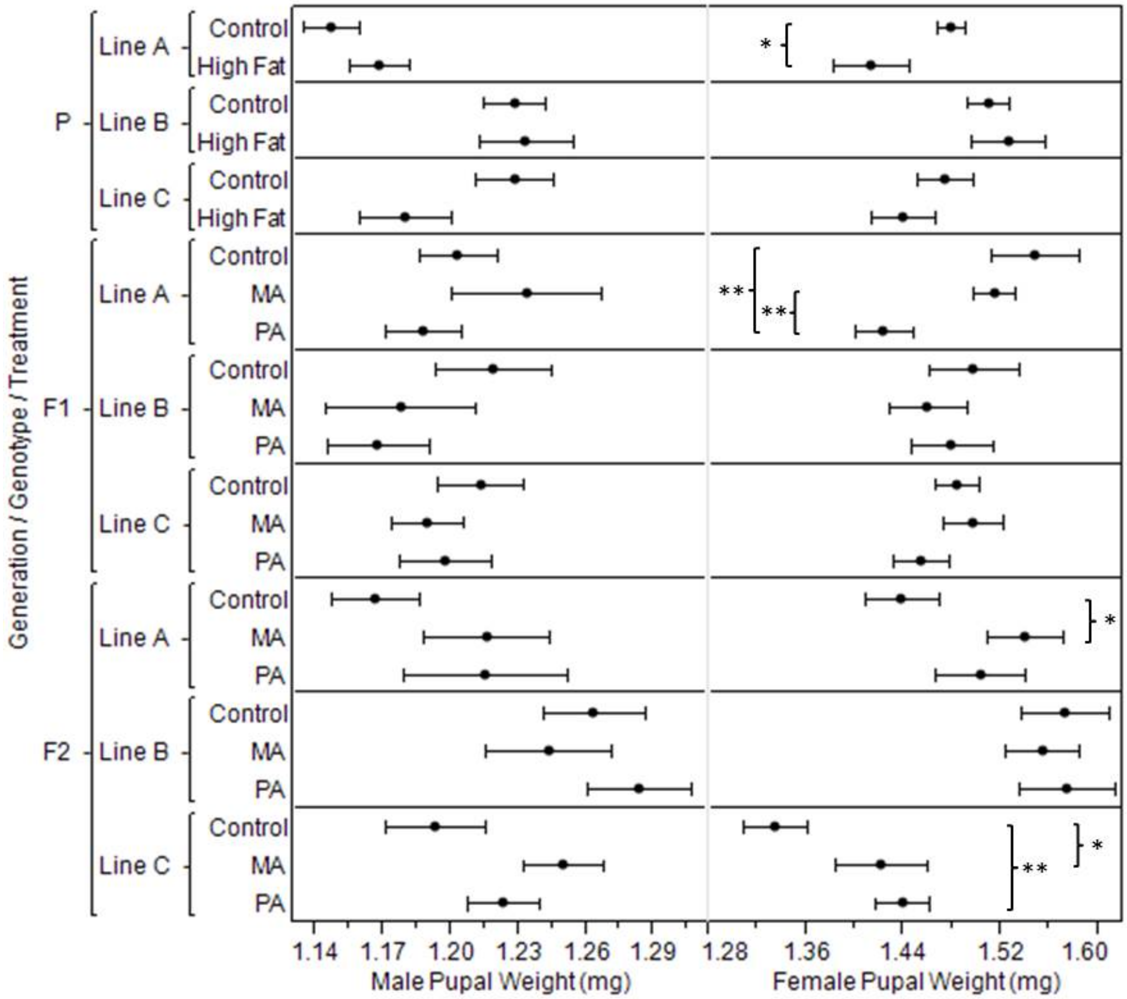

Figure F. Wet male and female pupal weight (mean ± SE) of Control, High Fat, Maternal Ancestor (MA), and Paternal Ancestor (PA) for three generations. Significance values indicated (p < 0.05 *, p < 0.01 **).

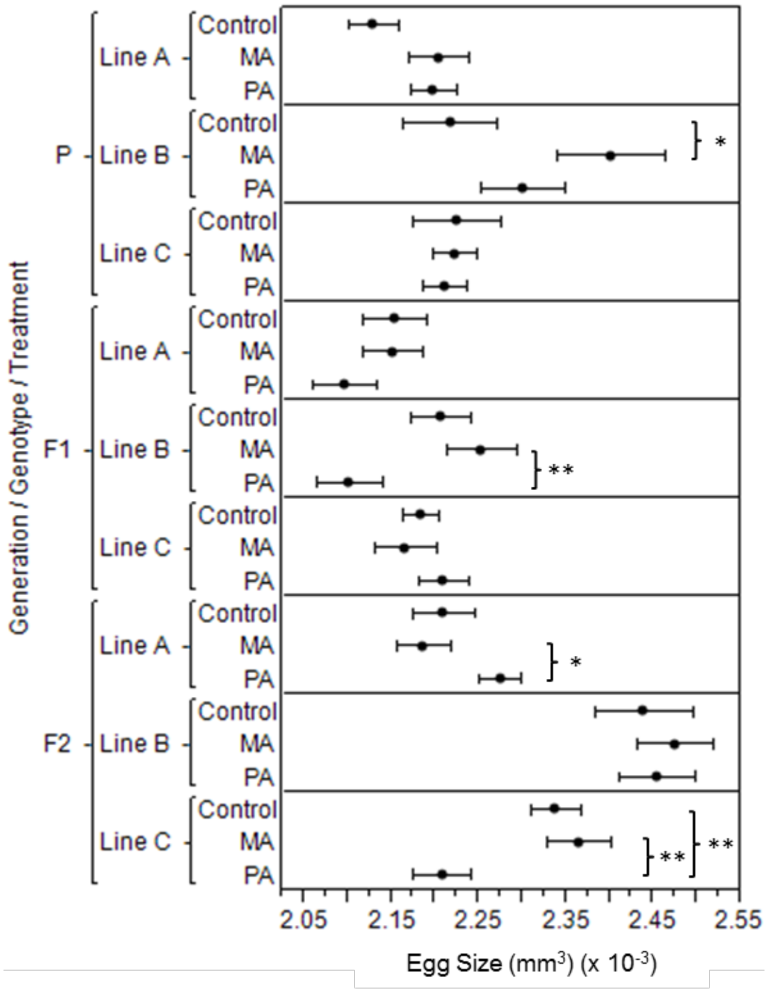

Figure G. Egg Size (mean ± SE) of Control, High Fat, Maternal Ancestor (MA), and Paternal Ancestor (PA) for three generations. Significance values indicated (*p* < 0.05 *, *p* < 0.01 **).

Table A. Parental generation variance partition for pupal weight.

Effect[1]	***10 line study*** variance explained[2]	NLP[3]	***4 line study*** variance explained[4]	NLP[3]
Genotype	0.2179	114.45	0.2595	134.90
Treatment	0.0001	*ns*	0.0166	14.92
Sex	0.5326	203.96	0.5278	207.49
Treatment*Genotype	0.0308	20.72	0.0036	2.65
Sex*Genotype	0.0149	9.05	0.0094	7.33
Treatment*Sex	0.0000	*ns*	0.0004	*ns*
Treatment*Sex*Genotype	0.0047	1.75	0.0003	*ns*
time replicate	*na*	*na*	0.0101	9.50

[1] ANOVA model effects, [2] variance in ***10 line study*** [3] negative log p-value; *ns,* non-significant, [4] variance in the ***4 line study***

Table B. Egg size and pupal weight correlations. Correlation reported as R^2-value with +/- sign representing the direction of significant correlations. Significant *p*-values reported parenthetically.

	Male Pupae				Female Pupae			
	Line A	Line B	Line C	Overall	Line A	Line B	Line C	Overa ll
Pupae → Egg Size [a]	0.078	+0.697 (**0.019**)	0.174	+0.498 (**0.0004**)	0.059	+0.754 (**0.011**)	-0.638 (**0.031**)	0.062
Egg size → Pupae [b]	0.001	-0.748 (**0.026**)	0.322	0.156	0.146	-0.668 (**0.047**)	0.518	0.276

[a] Tests if pupal weight correlates with the egg size of offspring
[b] Tests if egg size correlates with the future pupal weight
